# Do Curdlan Hydrogels Improved with Bioactive Compounds from Hop Exhibit Beneficial Properties for Skin Wound Healing?

**DOI:** 10.3390/ijms241210295

**Published:** 2023-06-18

**Authors:** Aleksandra Nurzynska, Katarzyna Klimek, Agnieszka Michalak, Katarzyna Dos Santos Szewczyk, Marta Arczewska, Urszula Szalaj, Mariusz Gagos, Grazyna Ginalska

**Affiliations:** 1 Chair and Department of Biochemistry and Biotechnology, Medical University of Lublin, Chodzki 1 Street, 20-093 Lublin, Poland; katarzyna.klimek@umlub.pl (K.K.); g.ginalska@umlub.pl (G.G.); 2 Independent Laboratory of Behavioral Studies, Medical University of Lublin, Chodzki 4 a Street, 20-093 Lublin, Poland; agnieszka.michalak@umlub.pl; 3 Department of Pharmaceutical Botany, Medical University of Lublin, Chodzki 1 Street, 20-093 Lublin, Poland; katarzyna.dossantos-szewczyk@umlub.pl; 4 Department of Biophysics, University of Life Sciences, Akademicka 13 Street, 20-033 Lublin, Poland; marta.arczewska@up.lublin.pl; 5 Department of Biophysics, Medical University of Lublin, Jaczewskiego 4 Street, 20-090 Lublin, Poland; 6 Laboratory of Nanostructures, Polish Academy of Science, Sokolowska 29/37 Street, 01-142 Warsaw, Poland; u.szalaj@labnano.pl; 7 Faculty of Materials Engineering, Warsaw University of Technology, 02-507 Warsaw, Poland; 8 Department of Cell Biology, Maria Curie-Sklodowska University, Akademicka 19, 20-033 Lublin, Poland; mariusz.gagos@poczta.umcs.lublin.pl; 9 Department of Biochemistry and Molecular Biology, Medical University of Lublin, Chodzki Street 1, 20-093 Lublin, Poland

**Keywords:** 1,3-β-d-glucan, hydrogel, wound dressing, xanthohumol, bitter acids, *Humulus lupulus* L., regenerative medicine, *Danio rerio*

## Abstract

Chronic wounds, among others, are mainly characterized by prolonged inflammation associated with the overproduction of reactive oxygen species and pro-inflammatory cytokines by immune cells. As a consequence, this phenomenon hinders or even precludes the regeneration process. It is known that biomaterials composed of biopolymers can significantly promote the process of wound healing and regeneration. The aim of this study was to establish whether curdlan-based biomaterials modified with hop compounds can be considered as promising candidates for the promotion of skin wound healing. The resultant biomaterials were subjected to an evaluation of their structural, physicochemical, and biological in vitro and in vivo properties. The conducted physicochemical analyses confirmed the incorporation of bioactive compounds (crude extract or xanthohumol) into the curdlan matrix. It was found that the curdlan-based biomaterials improved with low concentrations of hop compounds possessing satisfactory hydrophilicity, wettability, porosity, and absorption capacities. In vitro, tests showed that these biomaterials were non-cytotoxic, did not inhibit the proliferation of skin fibroblasts, and had the ability to inhibit the production of pro-inflammatory interleukin-6 by human macrophages stimulated with lipopolysaccharide. Moreover, in vivo studies showed that these biomaterials were biocompatible and could promote the regeneration process after injury (study on *Danio rerio* larvae model). Thus, it is worth emphasizing that this is the first paper demonstrating that a biomaterial based on a natural biopolymer (curdlan) improved with hop compounds may have biomedical potential, especially in the context of skin wound healing and regeneration.

## 1. Introduction

Chronic wounds (namely non-healing, most often infected surgical and traumatic wounds, venous ulcers, or diabetic foot ulcers) are a serious problem for society because they are associated with a decrease in the quality of life of patients, high costs of treatment, prolonged hospital stays, and, if such wounds are left untreated, can lead to sepsis and even death. Therefore, they require the use of bioactive dressings that will accelerate the healing process [1,2]. Wound dressings should meet several features to aid this healing process. Among other things, they should be non-cytotoxic and absorb excess wound exudate, as well as provide a moist environment, which, in turn, increases the rate of epithelialization. The most commonly used specialist dressings are polymer hydrogels, which, due to their chemical structure, provide an optimal moist environment and reduce the risk of maceration of the regenerated tissue when changing the dressing. Moreover, hydrogels constitute an ideal platform for the incorporation of bioactive compounds, such as antibiotics, antiseptics, and nanoparticles, etc., which allows them to increase their spectrum of biological activity [3,4,5,6,7].

β-glucans are an attractive heterogeneous group of polysaccharides that have the ability to form hydrogels. It has been demonstrated that such hydrogels can be modified by various compounds, which allows for their successful use in the fabrication of bioactive wound dressings [8,9,10,11,12]. For instance, Xiang et al. [10] developed injectable hydrogel composed of biguanide chitosan and oxidized β-glucan, which was additionally enriched by melanin nanoparticles. The authors showed that this biomaterial exhibited high antibacterial properties and possessed the ability to reduce bleeding and the production of reactive oxygen species (ROS), as well as pro-inflammatory cytokines, which indicates that it may be considered as a promising candidate for the treatment of chronic wounds. In turn, Pino et al. [12] fabricated a β-glucan/zinc oxide wound dressing with good antibacterial properties. It is also worth noting that there are few promising wound dressings based on curdlan (linear 1,3-β-d-glucan), which possess antibacterial properties. Wojcik et al. [13] developed biocompatible curdlan-based dressings enriched with gentamicin or zinc-doped nano-hydroxyapatite for the treatment of infected wounds. Michalicha et al. [6] fabricated poly(levodopa)-modified curdlan hydrogels that were additionally coupled with gentamicin. The authors indicated that such biomaterials stimulated clot formation, possessed high antibacterial activity, and were non-toxic towards Zebrafish larvae in vivo. On the other hand, Tong et al. [14] presented functionalized curdlan/polydopamine biomaterials enriched with chlorhexidine (CHX), which had antibacterial and cytocompatible properties.

Recently, our research team developed a hydrogel-like biomaterial based on curdlan and enriched with calcium ions for potential use as a dressing supporting skin wound healing [4]. This biomaterial has many beneficial physicochemical, structural, and biological properties. It possesses a porous structure, good absorbent properties, the ability to transmit water vapor, and the ability to release calcium ions, which are crucial for the wound healing process. Moreover, it is not only non-toxic, but also supports the viability and proliferation of human skin fibroblasts. It is also worth emphasizing that this biomaterial is fabricated by a simple, ion-exchanging dialysis method without the need to use drastic conditions (e.g., very high temperatures or harmful chemical compounds). This method enables its modification with sensitive natural and synthetic compounds in order to improve its biological activities (i.e., antibacterial, anti-inflammatory, antioxidant, and wound-healing properties) [4]. To continue our research into the development of polymeric curdlan-based dressing materials, we chose natural compounds derived from hop in order to modify this biomaterial. *Humulus lupulus* L. (hop) is a perennial dioecious plant that belongs to *Cannabaceae* [13]. The bioactive compounds from hop with therapeutic potential are mainly secondary metabolites, produced in the lupulin glands of female inflorescences. Secondary metabolites constitute 15–30% of the weight of the dried cones and they are divided into three main groups: (resinous) bitter acids (BA), essential oils, and polyphenols. Bitter acids, namely humulones (α-acids) and lupulones (β-acids), are commonly used in the food industry as bitter flavorings, but many studies have shown that BA can be also applied in medicine and pharmacy [15]. Both humulones and lupulones exhibit anti-cancer, anti-inflammatory, antioxidant, and antimicrobial properties, as well as affect the central nervous system (sedative, hypnotic activities) [16,17]. Importantly, Sleha et al. [18] reported that lupulones had a beneficial influence on skin wound healing in vivo (studies on porcine with methicillin-resistant *Staphylococcus aureus*-infected external wounds). The authors proved that β-acids exhibited strong antibacterial properties and also supported overall wound healing.

Apart from BA, polyphenols—especially xanthohumol (XN)—are also known to have a broad spectrum of biological activities. XN is a phenolic chalcone and its beneficial biological properties result from the presence of the α, β-unsaturated carbonyl group [19,20]. This polyphenol shows anti-inflammatory, antioxidant, anti-cancer, and antimicrobial activities [21,22,23]. Moreover, a few studies have also shown that xanthohumol possesses wound-healing properties [24,25,26,27]. For instance, Costa et al. [26] indicated that an oral administration of XN to diabetic Wistar rats decreased inflammation and oxidative stress, enabled proper neovascularization, and, as a consequence, supported the healing of diabetic wounds. Similarly, Lu et al. [27] demonstrated that XN decreased oxidative damage and promoted diabetic wound healing, thanks to the activation of nuclear factor erythroid 2-related factor 2 (Nrf2). Thus, they confirmed that XN can be considered as a promising agent in the treatment of diabetic skin ulcers. 

Taking into account the above reports, we hypothesized that a modification of the aforementioned curdlan-based biomaterial with compounds derived from hop (namely crude extract—CE, rich in α-acids, β-acids, and essential oils or pure xanthohumol—XN) will allow for the obtainment of a wound dressing with increased antibacterial, anti-inflammatory, and wound-healing properties. In order to verify this hypothesis, bioactive hop compounds were combined with curdlan-based biomaterials using ion-exchanging dialysis against calcium ions. The resultant hydrogels were subjected to evaluate their structural, physicochemical, and biological properties (i.e., antibacterial, cytocompatible, and anti-inflammatory activities in vitro, as well as biocompatibility towards zebrafish larvae in vivo).

To the best of our knowledge, this is the first study that assessed the importance of a biomaterial based on a natural biopolymer (curdlan) enriched with bioactive compounds derived from hop for the treatment of hard-to-heal chronic wounds. To date, only two papers [28,29] have shown the properties of biomaterials based on natural biopolymers (chitosan) that were modified with hop compounds. Nevertheless, the obtained chitosan-based biomaterials could be applicable as food packaging materials. The research proposed by our team will provide valuable insight into the potential biomedical applications of polysaccharide-based biomaterials enhanced with active hop components, which is particularly important due to the increasing antibiotic resistance [30,31,32], as well as the research trend that includes the fabrication of environmentally friendly biomaterials (Research trend included fabrication of environmentally friendly biomaterials are known. We are following this trend by production of biomaterials which contain natural ingredients.). 

## 2. Results and Discussion

### 2.1. Antioxidant and Anti-Inflammatory Activities of Hop Compounds

Biologically active compounds isolated from hops exhibit antioxidant and anti-inflammatory activities and they have been successfully studied as potential therapeutic agents. For instance, Liu et al. [33] showed their antioxidant properties and ability to scavenge excess amounts of reactive oxygen species (ROS), while Velot et al. [34] demonstrated the beneficial anti-inflammatory effects of hops extract in an in vitro model of osteoarthritis. Therefore, this study was carried out using crude extract—CE—and pure xanthohumol—XN—to establish whether they exhibit antioxidant and anti-inflammatory activities and, as a consequence, whether they can be used as modifiers of the curdlan matrix to produce biomaterials with antioxidant and anti-inflammatory properties.

The antioxidant activity was studied on the microplate scale in cell-free systems. The crude extract (CE) and xanthohumol (XN) from hop were evaluated in concentrations ranging from 0.00487 to 10 mg/mL and 0.0097 to 20 mg/mL, respectively. It was demonstrated that the crude extract, as well as the xanthohumol, exhibited moderate scavenging capacities in a concentration-dependent manner. A higher DPPH scavenging activity (Table S1) was shown for the CE (IC_50_ = 0.606 ± 0.011 mg/mL) when compared to XN (IC_50_ was 5.467 ± 0.065 mg/mL). For comparison, the IC_50_ value obtained at the same conditions for the positive control (standard), i.e., ascorbic acid (AA), was equal to 0.478 ± 0.02 mg/mL. The ABTS^●+^ assay (Table S1) revealed that both the crude extract from hop and xanthohumol possessed a similar ability to scavenge free radicals (IC_50_ = 0.486 ± 0.001 mg/mL and 0.638 ± 0.023 mg/mL, respectively). Nevertheless, these results were three times better than those obtained for the standard (AA), for which the IC_50_ was 1.988 ± 0.092 mg/mL. The results obtained in this study seem to be difficult to compare with the data provided in other publications, due to the other conditions used during these investigations. Nevertheless, the antioxidant activity using the DPPH assay was studied for a methanol extract from *Humulus japonicus*. Lee et al. [35] found that the studied extract possessed a scavenging effect with an IC_50_ value equal to 4.64 mg/mL. Thus, the result obtained in this study for the CE was much better compared to the data obtained by Lee et al. In turn, Lyu et al. [36] investigated the antioxidant activity of extracts from six different hop cultivars (Saaz, Calypso, Cascade, Cluster, El Dorado, and Magnum) using DPPH and ABTS assays. The El Dorado accession revealed the highest antioxidant activity in ethanol extracts (DPPH—the IC_50_ was approx. 0.124 mg/mL and for ABTS—the IC_50_ was approx. 0.193 µg/mL). In this case, both the CE and XN exhibited slightly inferior antioxidant activity compared to the data presented by Lyu.

The anti-inflammatory potential of the crude extract and xanthohumol was evaluated using in vitro assays investigating their inhibitory effects on pro-inflammatory enzymes, including cyclooxygenases (COX-1 and COX-2) and lipoxygenase (LOX). COX-1 (cyclooxygenase constitutive) is responsible for the maintenance of physiological prostanoid biosynthesis and COX-2 (inducible isoform) is connected to inflammatory tissues and cells, similar to lipoxygenases (LOXs) [37]. The crude extract and xanthohumol were tested at the same concentrations (10–200 µg/mL). Indomethacin (IND) for the COX assays and nordihydroguaiaretic acid (NDGA) for the lipoxygenase test were used as positive controls (standards). Both the CE and XN showed good activity against COX-1 and COX-2 (Table S2). In comparison to the CE, XN was found to be more active against COX-1 and COX-2, as well as against lipoxygenase, with IC_50_ values close to 20.54, 17.15, and 11.13 µg/mL, respectively.

It is worth underlining that extracts from hops contain large amounts of compounds with a high antioxidant potential, such as astragalin and rutin [38]. In addition to flavonoids, the compounds responsible for this antioxidant activity are β-acids [39]. It has been reported that a mixture of colupulone and n-lupulone plus ad-lupulone (1:4) possessed a higher antioxidant activity than colupolone alone [38]. In addition, Kontek et al. [40] demonstrated that a fraction rich in bitter acids, both humulones and lupulones, had a higher antioxidant potential than a second fraction rich in XN and α-acids.

It has also been proven that hop bitter acids and their derivatives possess anti-inflammatory activity (COX-2 inhibition) [41]. Furthermore, it has been shown that XN ingestion reduces inflammation, oxidative stress, and improves the wound healing process without toxicity in Wistar rats [24]. This compound has also shown the ability to regulate the activities of elastases/MMPs and stimulate the biosynthesis of fibrillar collagens, elastin, and fibrillins, preventing skin ageing [42]. In addition, xanthohumol can inhibit cytochrome P450 enzymes, which metabolically activate procarcinogens and induce pro-angiogenic pathways [38].

Thus, the results obtained within this study confirmed the data demonstrated by other authors and proved that both the CE (rich in α-acids, β-acids, and essential oils) and pure XN possess antioxidant and anti-inflammatory properties in vitro, which makes them promising compounds for the modification of biomaterials designed for the treatment of chronic wounds. In the process of wound healing, oxygen—which is the substrate for ATP—provides the necessary energy required for the regeneration of damaged tissues. Oxygen derivatives (O_2_ radicals)—ROS—are also important in the wound healing process, nevertheless, it is crucial to maintain a proper balance between reactive oxygen species and antioxidants [43]. This balance is referred to as redox homeostasis. Antioxidants nullify the damaging effects of ROS on cells by donating their electrons, thus preventing them from being captured by important biological molecules such as deoxyribonucleic acid (DNA). Under physiological conditions, cells produce ROS during metabolism, while their production is elevated under pathological conditions. In chronic wounds, which are characterized by prolonged inflammation (more than two weeks), inflammatory cells (mainly neutrophils) produce large amounts of ROS, which provide a defense against infection; nevertheless, an excess amount of ROS leads to the generation of oxidative stress, which, in turn, leads to the apoptosis of the cells surrounding the wound [44]. Thus, compounds that inhibit the overproduction of ROS and pro-inflammatory cytokines are promising for wound healing. Therefore, we have attempted to incorporate CE and XN into curdlan-based biomaterials. 

### 2.2. ATR-FTIR Spectra of Biomaterials

**Figure 1:**
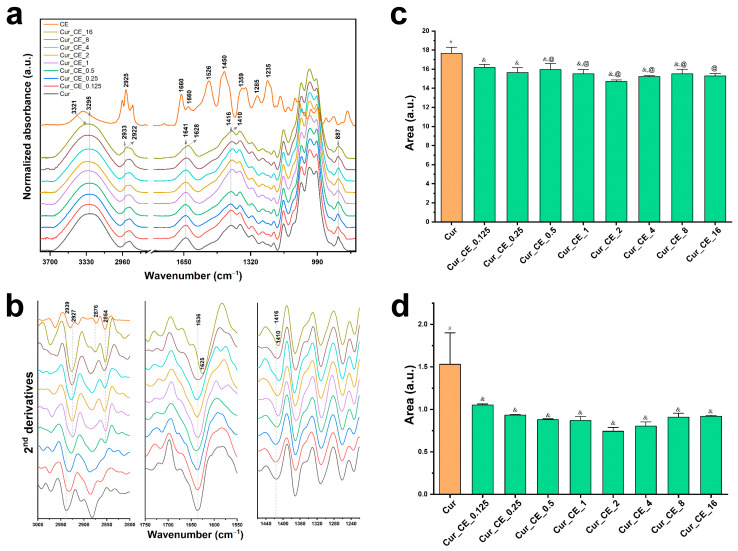
(**a**) Mean ATR-FTIR spectra (*n* = 4) of curdlan-based biomaterials (Cur) with different blend ratios (CE); (**b**) mean second-derivative spectra from the same samples presented in the regions of 3000−2800 cm^−8^, 1750−1580 cm^−5^, and 1445−1235 cm^−2^; (**c**) evolution of the integrated area of the OH bands in the stretching vibration region (3700−3000 cm^−0^), and (**d**) in the bending vibration region 1730−1580 cm^−5^ with different CE content in the biomaterial. (**a**,**b**) The spectra were offset along the y-axis for clarity. (**c**,**d**) Data are presented as mean ± SD, one-way ANOVA test followed by Tukey’s multiple comparison, different symbols indicate significantly different mean values (*p* < 0.05).

The FTIR spectra of the pure components (CE, curdlan) and curdlan-based biomaterials enriched with CE with different blend ratios are presented in Figure 1a. The second derivative spectra were performed to interpret the possible interactions between the components (Figure 1b). The crude extract (CE) mainly consisted of α-acids, β-acids, terpenes, and hop polyphenols. A very broad characteristic absorption peak in the CE spectrum, centered at about 3362 cm^−1^, originated from the stretching vibration of the –OH groups in the phenols. The bands within the 2800–3100 cm^−1^ range correspond to the antisymmetric and symmetric stretching vibrations of both the methyl and methylene groups, which could also be found in polysaccharides. The presence of aromatic structures was established by the C=C skeleton vibration bands between 1690 and 1400 cm^−1^, with most of these bands being associated with the phenyl rings [45,46,47]. Moreover, a low intensity, which is visible at 1628 cm^−1^, could be linked to the stretching vibration of the carbonyl group coming from the different polyphenyl derivatives present in the hop extract. The C–O and C–O–C group absorption peaks appeared at 1000 and 1200 cm^−1^ (Figure 1a). 

In the case of the curdlan-based biomaterial, the stretching vibration band of the hydrogen-bonded hydroxyl groups appeared at 3295 cm^−1^, the absorption band of the CH_3_ group could be observed at 2933 cm^−1^, and the bending vibrations in the water molecules (H–O–H) at 1641 cm^−1^ [38,39]. The absorption bands at 1416 and 1372 cm^−1^ originated from the bending modes of CH_2_, CH, and OH. Although an accurate assignment of the polysaccharide spectra remains problematic, each particular polysaccharide had a specific band maximum in the 1300–800 cm^−1^ region [48]. The intense overlapped bands in this region related to the C–O, C–C stretching and C–OH bending modes [49]. More importantly, the characteristic band of the β- (1 → 3) glucosidic linkage bond and β conformers can be clearly distinguished at 887 cm^−1^ [33].

After the modification of the curdlan-based biomaterials with the CE, there was a narrowing and 26 cm^−1^ shift of the OH band to higher wavenumbers as the crude extract was added at 1–16 μg/mL. Because this band is a sensitive indicator of hydrogen bond strength, its shift is attributed to the weakened hydrogen bonding between the curdlan chains [50]. On the other hand, in comparison to the control biomaterial, the following bands at 2933, 1640, and 1416 cm^−1^ were slightly shifted to lower wavenumbers for the curdlan-based biomaterial enriched with the CE. Moreover, the broadening of an anomeric region (900−800 cm^−1^) was observed (Figure 1a). Previous research has shown that the red-shift of these bands indicates that more hydrogen bonds were formed [51,52]. The intermolecular interactions via the hydrogen bonds between Cur and CE in a concentration of above 0.5 μg/mL might contribute to the decreased hydrophilic nature of the control biomaterial by reducing the free hydrophilic sites [53]. A second derivative of the spectra in the mentioned regions was performed to better visualize the changes noted (Figure 1b), and the integrated area of the bands characteristic for the OH stretching and bending vibrations were calculated (Figure 1c). The most distinguishing changes in the second derivative spectra of the curdlan-CE modified biomaterial were related to the shift of the band originating from the CH_2_ asymmetric stretching vibrations to the lower wavenumber, in comparison to Cur (from 2939 to 2927 cm^−1^ in higher concentrations of CE) and CE (from 2929 to 2927 cm^−1^). Furthermore, in the curdlan-based biomaterials, a new band at 2854 cm^−1^, corresponding to the C-H stretching, appeared in the same region as in the CE spectrum (Figure 1b). This was especially noticeable in the spectra of Cur_CE_0.5–Cur_CE_16 and presents further proof of the good incorporation of the bioactive hop compounds into the curdlan matrix. Intra- and intermolecular hydrogen bond interactions between curdlan and incorporated active molecules have been reported in the literature as well [54,55,56]. For example, Zhou et al. found that tea polyphenols incorporated into curdlan/chitosan blended films significantly increased their mechanical strength through a reduction in the polysaccharides’ chain mobility [54]. The gradual spectral shift of the band at 1416 cm^−1^, representing the C-OH deformation vibrations in Cur towards lower frequencies (to 1410 cm^−1^ in the most CE-enriched samples), suggests the involvement of the hydroxyl groups of the crude extract compounds in the binding via the H-bond to the curdlan-based biomaterials (Figure 1b). The reduction in the integral area of the OH band in the stretching (Figure 1c) and bending vibration region (Figure 1d), as compared to the control sample, may be related to the decrease in the availability of the hydroxyl groups in the CE-modified biomaterial.

**Figure 2:**
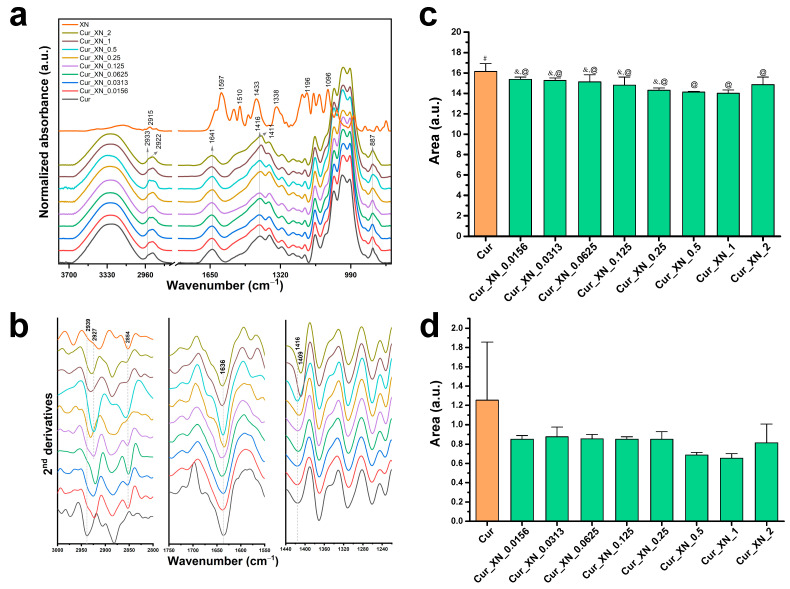
(**a**) Mean ATR-FTIR spectra (*n* = 4) of curdlan-based biomaterials (Cur) with different blend ratios (XN); (**b**) mean second-derivative spectra from the same samples presented in the regions of 3000−2800 cm^−8^, 1750−1580 cm^−5^, and 1445−1235 cm^−2^; (**c**) evolution of the integrated area of the OH bands in the stretching vibration region (3700−3000 cm^−0^), and (**d**) in the bending vibration region 1730−1580 cm^−5^ with different XN content in the biomaterial. The spectra were offset along the y-axis for clarity. (**c**,**d**) Data are presented as mean ± SD, one-way ANOVA test followed by Tukey’s multiple comparison, different symbols indicate significantly different mean values (*p* < 0.05). In the case of integrated intensity in the region of 1730−1580 cm^−5^, the difference of the means is not significant (*p* > 0.05).

Similar results were obtained in the case of the study of the possible chemical interactions between the functional groups of the components in the curdlan-based biomaterials enriched with XN. Figure 2 presents the FTIR spectra of the pure components (XN, curdlan) and curdlan-based biomaterials with XN in different blend ratios. The FTIR spectrum of the XN powder reveal the main characteristic absorption bands, which are similar to the spectra presented elsewhere [47,57]. The incorporation of XN into the Cur biomaterial resulted in a slight shift of the bands at 2933 and 1416 cm^−1^ to lower wavenumbers as the XN concentration increased. Unlike previous investigations, no shift of the band at 1640 cm^−1^ and a broadening of the region related to the β-(1,3)-glucan linkages were observed upon the curdlan modification (Figure 2a). The second derivative spectra reveal the changes within the region of the C-H stretching vibration in the case of all the XN concentrations, while a red-shift of the band at 1416 cm^−1^, representing the deformation vibration of the CH_2_ in the glycoses, was noticeable for the highest concentrations of XN (Cur_XN_1 and Cur_XN_2); see Figure 2b. 

Figure 2c,d show the integrated area of the OH absorption bands in the stretching and bending vibration region. Moreover, in contrast to the curdlan-based biomaterials enriched with CE, a significant reduction in the integral area under the OH band involved only the stretching vibrations region (Figure 1c). The increased intensity of this band could confirm the hydrophobic nature of the curdlan-based biomaterials enriched with both the crude extract and xanthohumol discussed in Section 2.4.

### 2.3. SEM Images and EDS Spectra of Biomaterials

**Figure 3:**
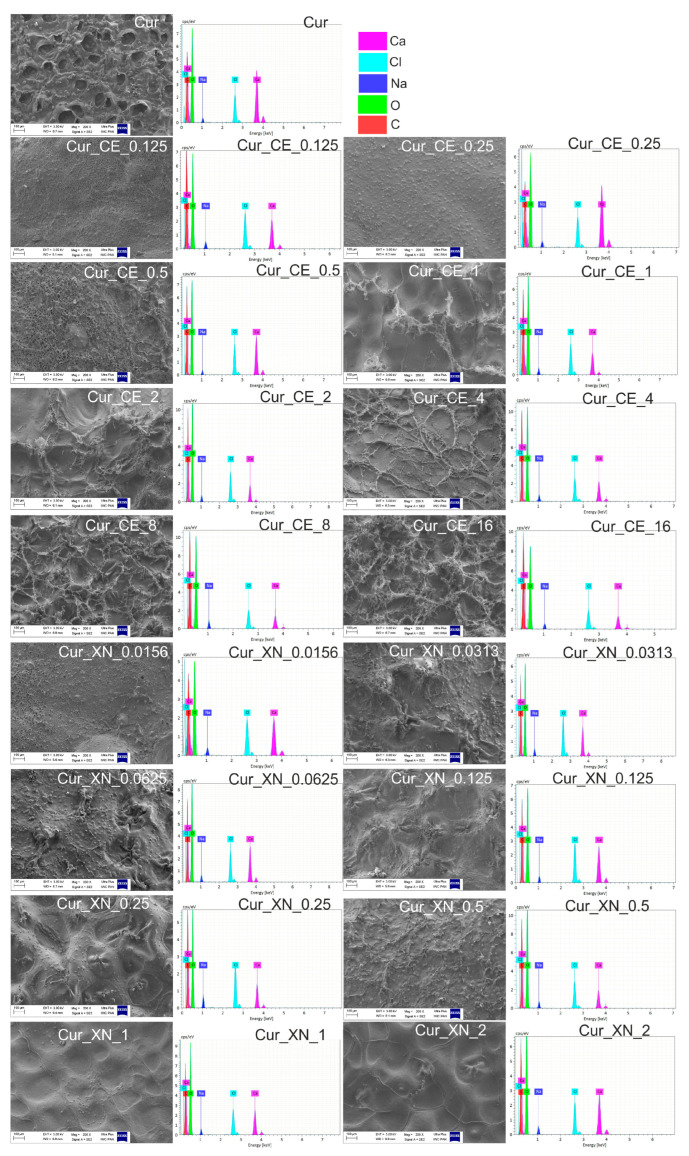
SEM images and EDS spectra of curdlan-based biomaterials improved with crude extract (CE) and xanthohumol (XN). SEM magnification = 200×, scale bar = 100 μm.

An SEM analysis (Figure 3) showed that the biomaterials enriched with the CE (Cur_CE_0.125–Cur_CE_16) and XN (Cur_XN_0.0156–Cur_XN_2) had smaller pores compared to the control biomaterial (Cur), which may indicate that the bioactive hop compounds filled the pores of the biomaterial. Importantly, the addition of CE and XN to the curdlan-based biomaterial did not cause any cracking or deformation of the biomaterials. EDS spectra also proved that the incorporation of bioactive substances did not change the elemental composition of the biomaterials (Figure 3).

### 2.4. Contact Angle of Biomaterials

**Figure 4:**
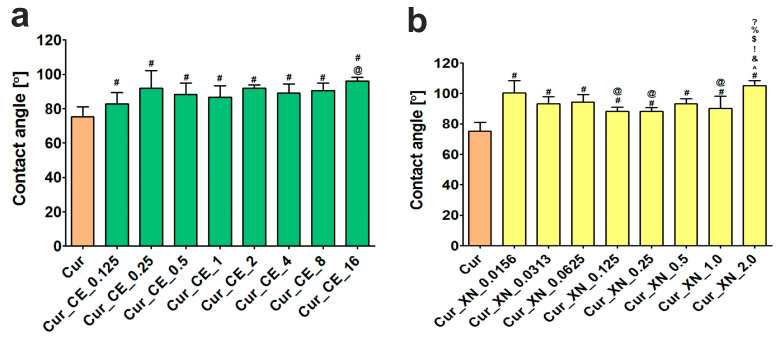
Wettability of the surface of curdlan-based biomaterials improved with crude extract (CE) (**a**) and xanthohumol (XN) (**b**). ^#^ Significantly different results compared to results obtained for control biomaterial (Cur); ^@^ significantly different results compared to results obtained for Cur_CE_0.125 biomaterial (**a**) or Cur_XN_0.0156 biomaterial (**b**); ^^^ significantly different results compared to results obtained for Cur_XN_0.0313 biomaterial (**b**); ^&^ significantly different results compared to results obtained for Cur_XN_0.0625 biomaterial (**b**); ^!^ significantly different results compared to results obtained for Cur_XN_0.125 biomaterial (**b**); ^$^ significantly different results compared to results obtained for Cur_XN_0.25 biomaterial (**b**); ^%^ significantly different results compared to results obtained for Cur_XN_0.5 biomaterial (**b**); ^?^ significantly different results compared to results obtained for Cur_XN_1 biomaterial (**b**); one-way ANOVA test followed by Tukey’s multiple comparisons, *p* < 0.05.

The measurements of the contact angles indicated that the surfaces of the curdlan-based biomaterials enriched with both the crude extract (Figure 4a) and xanthohumol (Figure 4b) possessed a significantly lower wettability compared to the surface of the control curdlan biomaterial (Cur). It has been established that biomaterial surfaces characterized by contact angles lower than 90° are considered to be hydrophilic, while those with contact angles ranging from 90° to 180° are considered to be hydrophobic, respectively [58]. Thus, considering our results, it should be underlined that only the surfaces of the control biomaterial (Cur) and Cur_CE_0.125 biomaterial (Figure 4a) had a hydrophilic nature (contact angles equal to 75.30 ± 5.75 and 82.68 ± 5.51, respectively). In turn, the contact angles for all the other biomaterials were over 90° (Figure 4a,b), which indicates that their surfaces were hydrophobic. These results are in good agreement with the data provided by Bajić et al. [29], as they indicated that the compounds from the hop possessed a hydrophobic nature.

### 2.5. Swelling Ability of Biomaterials

**Figure 5:**
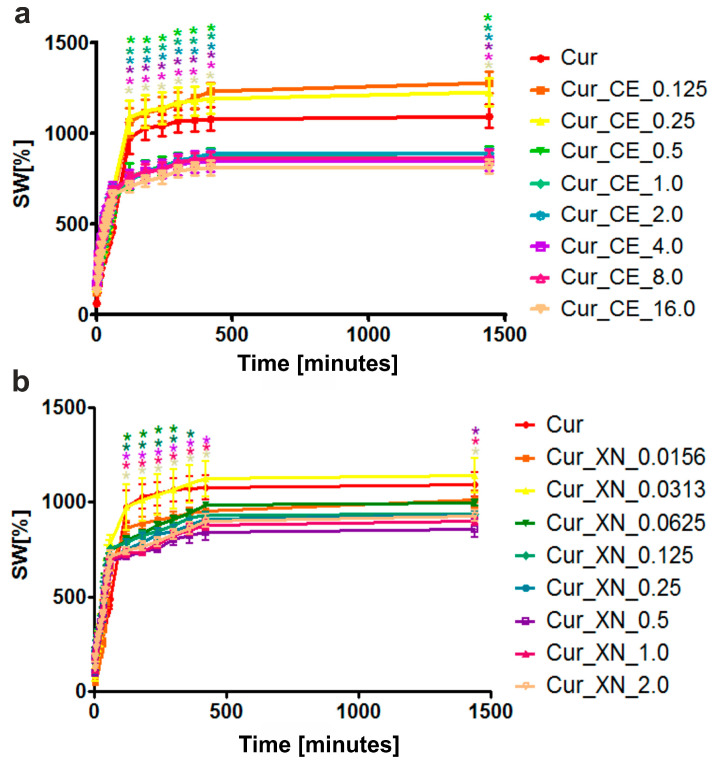
The ability of curdlan-based biomaterials enriched with (**a**) crude extract (CE) and (**b**) xanthohumol (XN) to absorb simulated wound fluid (SWF) after 24 h of incubation. * Statistically significant differences compared to control curdlan-based biomaterial (Cur), according to a Two-Way ANOVA test, followed by a Bonferroni comparison test, *p* < 0.05.

The absorption test showed that the higher the concentration of the bioactive compounds (CE and XN) in the curdlan-based biomaterials, the lower their ability to swell in the presence of SWF (Figure 5a,b). Nevertheless, in the case of the biomaterials with low concentrations of bioactive compounds, i.e., Cur_CE_0.125, Cur_CE_0.25, Cur_XN_0.0156, Cur_XN_0.0313, and Cur_XN_0.0625, no significant differences (*p* > 0.05) in the amount of absorbed SWF were observed in comparison to the control biomaterial (Cur). In turn, the other biomaterials swelled significantly less beginning from the 120th minute of incubation. Most probably, this decreased ability to swell resulted from the lower porosity of the biomaterials enriched with bioactive hop components compared to the control curdlan-based biomaterial, as proven by the SEM images (Figure 3), as well as the fact that the surfaces of the modified biomaterials were hydrophobic (Figure 4a,b). Interestingly, our results are in good agreement with the data presented by Bajić et al. [29]. The authors demonstrated that the chitosan-based films enriched with hop extract possessed a lower ability to absorb liquid compared to the control chitosan-based biomaterial and they proposed two scenarios underlying this phenomenon. The first of them involved the occurrence of interactions between the chitosan and hop compounds, which led to the blocking of the functional groups of the polymer, which are responsible for its ability to absorb liquids. In turn, the second reason was related to the hydrophobic nature of the tested hop extract. 

The ability of biomaterials to absorb liquid is one of the important features of potential wound dressings. Chronic wounds are accompanied by a high amount of wound exudates, and absorbent biomaterials are thus crucial for the treatment of such ailments [3,4]. In this study, we proved that the addition of bioactive compounds (either CE or XN) to a curdlan-based biomaterial decreased the swelling ability of the resultant biomaterials. Nevertheless, the ability of the biomaterials enriched with the CE and XN to absorb SWF was still high. Even the biomaterials loaded with the highest amount of hop compounds (Cur_CE_16 and Cur_XN_2) had the ability to increase their weight by approx. 800% compared to their weight in a dry state. Therefore, all the tested biomaterials possessed acceptable absorbent properties. 

### 2.6. Antibacterial Activity of Biomaterials

**Figure 6:**
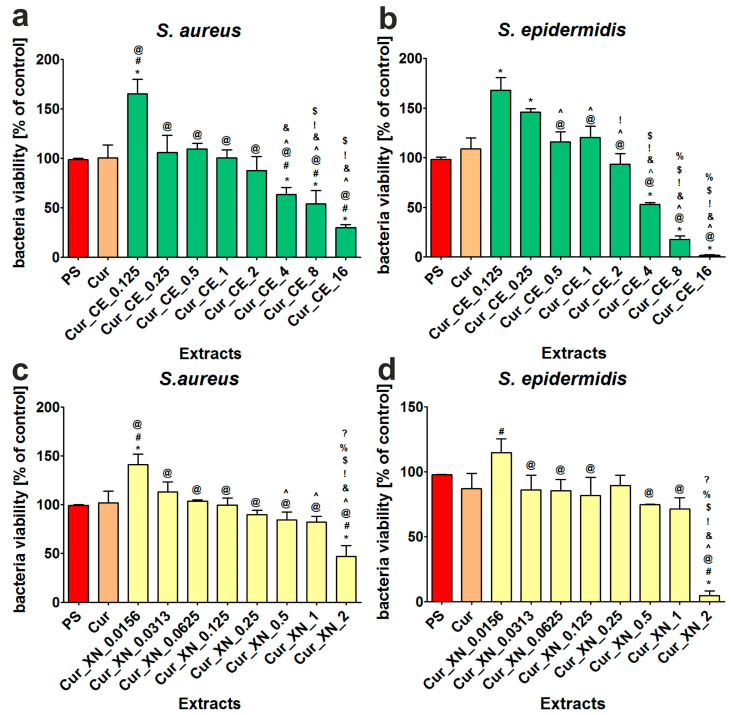
Antibacterial activity of extracts obtained from curdlan-based biomaterials improved with crude extract (CE) (**a**,**b**) and xanthohumol (XN) (**c**,**d**). * Significantly different results compared to results obtained for extracts from polystyrene (PS)—control of the experiment. ^#^ Significantly different results compared to results obtained for extracts from control biomaterial (Cur); ^@^ significantly different results compared to results obtained for extracts from Cur_CE_0.125 biomaterial (**a**,**b**) or Cur_XN_0.0156 biomaterial (**c**,**d**); ^^^ significantly different results compared to results obtained for extracts from Cur_CE_0.25 (**a**,**b**) or Cur_XN_0.0313 biomaterial (**c**,**d**); ^&^ significantly different results compared to results obtained for extracts from Cur_CE_0.5 (**a**,**b**) or Cur_XN_0.0625 biomaterial (**c**,**d**); ^!^ significantly different results compared to results obtained for extracts from Cur_CE_1 (**a**,**b**) or Cur_XN_0.125 biomaterial (**c**,**d**); ^$^ significantly different results compared to results obtained for extracts from Cur_CE_2 (**a**,**b**) or Cur_XN_0.25 biomaterial (**c**,**d**); ^%^ significantly different results compared to results obtained for extracts from Cur_CE_4 (**a**,**b**) or Cur_XN_0.5 biomaterial (**c**,**d**); ^?^ significantly different results compared to results obtained for extracts from Cur_CE_8 (**a**,**b**) or Cur_XN_1 biomaterial (**c**,**d**); one-way ANOVA test followed by Tukey’s multiple comparison, *p* < 0.05.

Individual hop compounds, as well as various hops extracts, are known for their antimicrobial properties [59]. Our previous research has shown that both CE and XN have growth-inhibiting properties against Gram-positive bacteria [21]. In this study, the antibacterial activity of curdlan-based biomaterials enriched with CE and XN was evaluated. The experiments performed with the use of liquid extracts indicated that the tested biomaterials had antibacterial activity depending on the concentration of the incorporated hop compounds (Figure 6a–d). Thus, it was observed that only extracts from the biomaterials enriched with high concentrations of hop compounds were able to significantly inhibit (*p* < 0.05) the growth of *S. aureus* and *S. epidermidis* in comparison to the control extracts (PS and Cur). In the case of the curdlan-based biomaterials improved with CE, the growth of *S. aureus* was inhibited to approx. 63% (extract from Cur_CE_4 biomaterial), 54% (extract from Cur_CE_8 biomaterial), and 30% (extract from Cur_CE_16 biomaterial), while the growth of *S. epidermidis* was reduced to approx. 53% (extract from Cur_CE_4 biomaterial), 18% (extract from Cur_CE_8 biomaterial), and 1% (extract from Cur_CE_16 biomaterial), respectively. In turn, the curdlan-based biomaterial enriched with XN at a concentration of 2 mg/mL (Cur_XN_2) exhibited a significant inhibition (*p* < 0.05) of *S. aureus* and *S. epidermidis* growth to approx. 47% and 5% compared to the control extract, respectively. 

Many authors have suggested that α- and β-acids, which were identified in large amounts in our crude extract (CE), are mainly responsible for the antimicrobial properties of hops. For example, Michiu et al. proved the inhibitory effects of hop iso-α and -β acids against the bacterium *Pediococcus pentosaceus* at both high and low pH values [60]. Moreover, Cermak and co-authors reported the antimicrobial activity of humulone, lupulone, and xanthohumol against some anaerobic bacteria of indigenous human flora, and their study indicated that xanthohumol showed the highest antimicrobial activity against all these bacteria, followed by β-acids and α-acids [61]. Hop’s terpenes have also shown moderate antimicrobial effects against Gram-negative and Gram-positive bacteria [38]. Additionally, a hop CO_2_ extract with 50% humulone and lupulone exhibited high antibacterial activity against *Propionibacterium acnes* and *Staphylococcus aureus* [62].

It is worth noting that the main mechanism of the antibacterial activity of wound dressings involves the release of active antibacterial agents (e.g., antibiotics, antiseptics, and metal nanoparticles, etc.) into the surrounding liquid environment (namely in contact with wound exudate), which allows the prevention of bacteria colonization and growth at the wound bed [63,64]. Therefore, our results demonstrated that curdlan-based biomaterials enriched with hop compounds (i.e., Cur_CE_4 and Cur_XN_2 biomaterials) can potentially be considered as dressings that inhibit the growth of bacteria in the wound bed.

### 2.7. Cell Culture Experiments In Vitro

Dressings enriched with natural bioactive compounds for the healing of chronic wounds should not exhibit cytotoxicity, nor should they inhibit skin cell proliferation. In light of the above, it is crucial to determine which concentration of a bioactive compound incorporated into a biomaterial will not exhibit cytotoxic effects and, at the same time, will have antioxidant and anti-inflammatory effects, accelerating the healing process. Accordingly, in vitro studies were conducted to determine the optimal concentration of hop compounds in curdlan-based biomaterials and thus select the most promising candidates as wound dressings.

#### 2.7.1. The Influence of Biomaterials on the Viability and Proliferation of Human Fibroblasts

**Figure 7:**
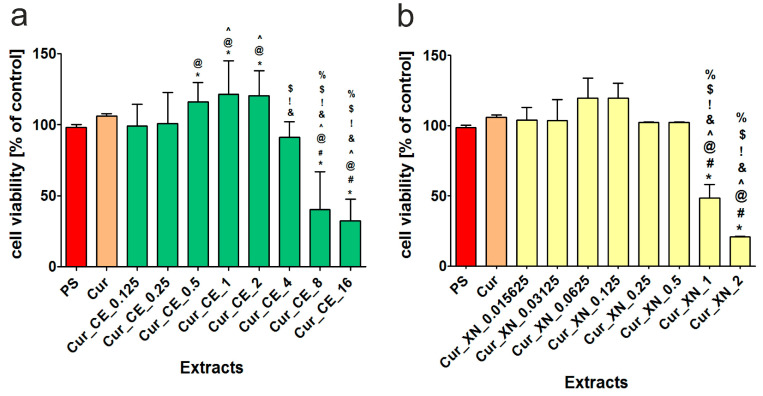
Viability of normal human fibroblasts (BJ cell line) after 24 h of incubation with extracts obtained from curdlan-based biomaterials enriched with CE (**a**) and XN (**b**). Extracts obtained from polystyrene (PS) and non-modified curdlan-based biomaterial (Cur) served as controls. * Significantly different results compared to results obtained for extracts from polystyrene (PS)—control of the experiment. ^#^ Significantly different results compared to results obtained for extracts from control biomaterial (Cur); ^@^ significantly different results compared to results obtained for extracts from Cur_CE_0.125 biomaterial (**a**) or Cur_XN_0.0156 biomaterial (**b**); ^^^ significantly different results compared to results obtained for extracts from Cur_CE_0.25 (**a**) or Cur_XN_0.0313 biomaterial (**b**); ^&^ significantly different results compared to results obtained for extracts from Cur_CE_0.5 (**a**) or Cur_XN_0.0625 biomaterial (**b**); ^!^ significantly different results compared to results obtained for extracts from Cur_CE_1 (**a**) or Cur_XN_0.125 biomaterial (**b**); ^$^ significantly different results compared to results obtained for extracts from Cur_CE_2 (**a**) or Cur_XN_0.25 biomaterial (**b**); ^%^ significantly different results compared to results obtained for extracts from Cur_CE_4 (**a**) or Cur_XN_0.5 biomaterial (**b**); one-way ANOVA test followed by Tukey’s multiple comparison, *p* < 0.05.

After 24 h of incubation, the MTT assay indicated that the extracts obtained from the curdlan-based biomaterials enriched with CE (Figure 7a) and XN (Figure 7b) showed various effects on cell viability, depending on the concentration of the incorporated hop compounds. Thus, it is indicated that the extracts obtained from Cur_CE_0.5, Cur_CE_1, and Cur_CE_2 significantly increased (*p* < 0.05) the viability of BJ cells compared to both control extracts (PS, Cur). In turn, the extracts obtained from the biomaterials enriched with the highest concentrations of CE (namely Cur_CE_8 and Cur_CE_16) significantly reduced the viability of skin fibroblasts, which indicates that these biomaterials should be considered cytotoxic towards eukaryotic cells. In the case of the curdlan-based biomaterials improved with XN (Figure 7b), it was observed that the extracts from Cur_XN_0.0625 and Cur_XN_0.125 increased the viability of skin fibroblasts, but these results were not statistically significant (*p* > 0.05) compared to the data obtained for the extracts from the control biomaterials (PS, Cur). The extracts from the biomaterials improved with XN in the highest concentrations (Cur_XN_1 and Cur_XN_2) caused significant cytotoxic effects towards skin fibroblasts, which limits their potential application in the biomedical sector. 

**Figure 8:**
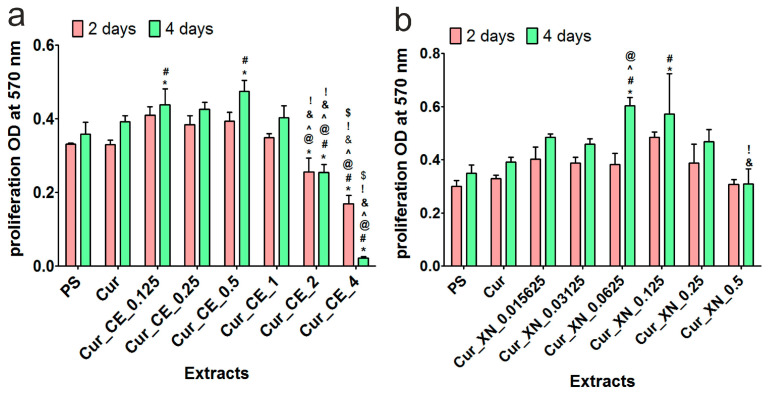
The proliferation of normal human fibroblasts (BJ cell line) after 2 and 4 days of incubation with extracts obtained from curdlan-based biomaterials enriched with CE (**a**) and XN (**b**). Extracts obtained from polystyrene (PS) and non-modified curdlan-based biomaterial (Cur) served as controls. * Significantly different results compared to results obtained for extracts from polystyrene (PS)—control of the experiment. ^#^ Significantly different results compared to results obtained for extracts from control biomaterial (Cur); ^@^ significantly different results compared to results obtained for extracts from Cur_CE_0.125 biomaterial (**a**) or Cur_XN_0.0156 biomaterial (**b**); ^^^ significantly different results compared to results obtained for extracts from Cur_CE_0.25 (**a**) or Cur_XN_0.03125 (**b**); ^&^ significantly different results compared to results obtained for extracts from Cur_CE_0.5 (**a**) or Cur_XN_0.0625 biomaterial (**b**); ^!^ significantly different results compared to results obtained for extracts from Cur_CE_1 (**a**) or Cur_XN_0.125 biomaterial (**b**); ^$^ significantly different results compared to results obtained for extracts from Cur_CE_2 (**a**); two-way ANOVA test followed by a Bonferroni comparison, *p* < 0.05.

Considering the data obtained during the cytotoxicity evaluation (Figure 7a,b), to assess the effect of the biomaterial extracts on fibroblast proliferation, only those that did not significantly reduce cell viability were selected. It was found that most of the extracts obtained from the curdlan-based biomaterials enriched with CE (Figure 8a) did not inhibit the proliferation of BJ cells compared to the control extracts (PS, Cur). Importantly, after 4 days of incubation, the extracts from Cur_CE_0.125 and Cur_CE_0.5 significantly promoted (*p* < 0.05) the divisions of skin fibroblasts in comparison to the control extracts, which indicates that these biomaterials can potentially have a positive effect on the wound healing process. Unfortunately, the extracts from Cur_CE_2 and Cur_CE_4 significantly inhibited (*p* > 0.05) the proliferation of BJ cells compared to both control extracts, as well as the extracts obtained from the other modified biomaterials. In turn, all the tested extracts from the curdlan-based biomaterials enriched with XN (Figure 8b) not only did not inhibit BJ cell division compared the control extracts (PS and Cur), but two of them (Cur_XN_0.0625 and Cur_XN_0.125) also significantly enhanced the proliferation of these cells (*p* < 0.05, after 4 days of incubation). 

**Figure 9:**
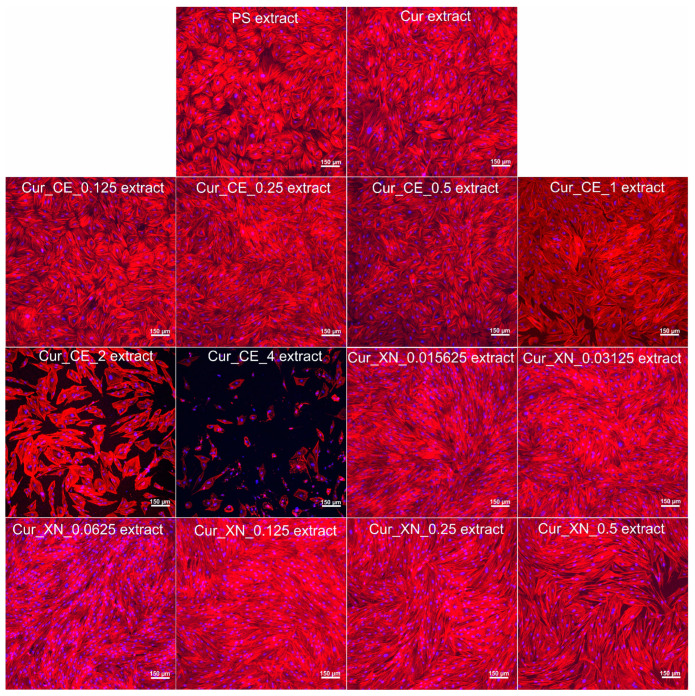
Morphology of human skin fibroblasts after 4 days of incubation with selected extracts of curdlan-based biomaterials enriched with CE and XN. Extracts from polystyrene (PS) and non-modified curdlan-based biomaterial (Cur) served as controls. Cell nuclei were stained with Hoechst 33342 dye (shown as blue fluorescence) and actin filaments of the cytoskeleton were stained with AlexaFluor^TM^ 635 Phalloidin dye (shown as red fluorescence). Images were obtained using a confocal laser scanning microscope (Olympus Fluoview equipped with FV1000, Shinjuku, Japan); magnification × 100, scale bar 150 μm.

In order to verify the results obtained during the spectrophotometric assay (Figure 8a,b), after 4 days of incubation, cell nuclei and actin filaments were additionally visualized (Figure 9). CLSM observations confirmed the data obtained with the MTT assay. It was shown that most of the tested extracts did not influence the morphology of human skin fibroblasts. In the case of the extracts obtained from the Cur_CE_2 and Cur_CE_4 biomaterials, changes in the cell number and shape were visible (Figure 9), which is in good agreement with previous results (Figure 8a), as the extracts from these biomaterials significantly reduced the metabolic activity of cells. On the other hand, none of the extracts obtained from the curdlan-based biomaterials enriched with XN had an influence on the BJ cell morphology (Figure 9). Interestingly, to date, hop compounds (humulones, lupulones, and polyphenols) have been studied mainly as anti-cancer agents. Thus, many papers have indicated that they possess the ability to inhibit the proliferation of various cancer cells [65,66,67,68,69]. Our results indicated that both pure xanthohumol at low concentrations and crude extracts rich in α-acids, β-acids, and essential oils can be considered as promising ingredients for biomaterials designed for supporting the healing process.

#### 2.7.2. The Influence of Biomaterials on the Production of IL-6 by LPS-Stimulated Human Macrophages

**Figure 10:**
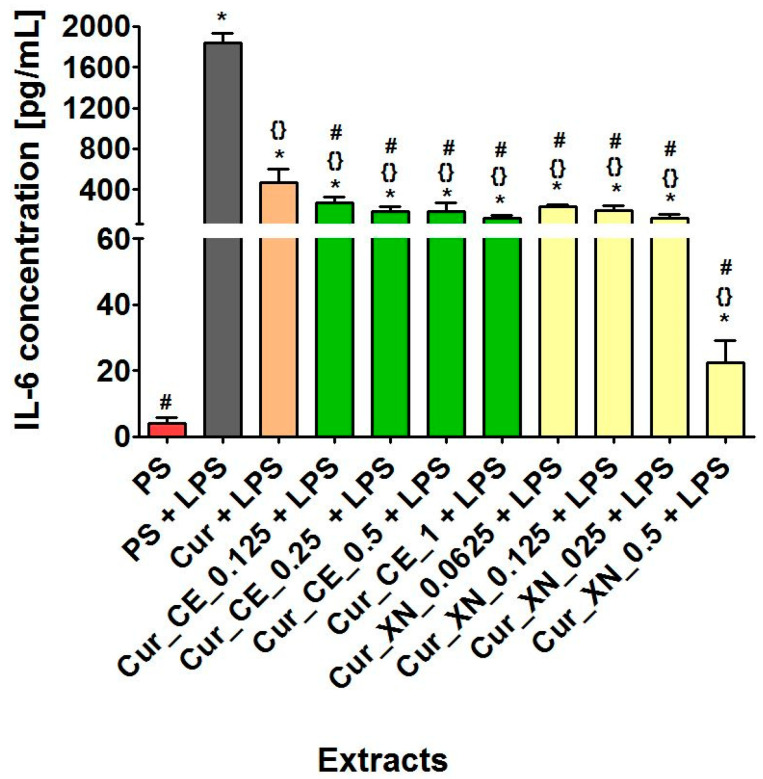
The concentration of IL-6 synthesized by THP-1-derived macrophages after 24 h of incubation with selected extracts obtained from curdlan-based biomaterials enriched with CE or XN. Extract from polystyrene (PS) served as a negative control, while the extract from PS with the addition of 100 ng/mL LPS served as a positive control. In order to determine the anti-inflammatory properties of extracts from tested curdlan-based biomaterials, they were also supplemented with 100 ng/mL LPS. The data were obtained using ELISA assay. * Significantly different results compared to results obtained for extracts from polystyrene (PS)—negative control of the experiment. ^{}^ Significantly different results compared to results obtained for extracts from polystyrene (PS) supplemented with 100 ng/mL LPS—positive control of the experiment. ^#^ Significantly different results compared to results obtained for extracts from control biomaterial (Cur) supplemented with 100 ng/mL LPS; one-way ANOVA test followed by Tukey’s multiple comparisons, *p* < 0.05.

Based on data obtained during the previous cell culture experiments (Figure 7a,b, Figure 8a,b and Figure 9), only the most promising extracts from the curdlan-based biomaterials were subjected to an evaluation of their anti-inflammatory properties. The ELISA test indicated that all the tested extracts from the biomaterials significantly decreased the production of IL-6 by THP-1-derived macrophages after LPS stimulation (Figure 10). Interestingly, even the extract from the control biomaterial (Cur + LPS) had the ability to inhibit the inflammatory response of LPS-induced macrophages in comparison to the stimulated extract obtained from the polystyrene-positive control (PS + LPS). Moreover, all the tested extracts from the curdlan-based biomaterials enriched with CE or XN possessed a significant ability to decrease the IL-6 production by THP-1-derived macrophages compared to both the simulated PS extract (PS + LPS) and simulated curdlan-based extract (Cur + LPS), which may indicate that the hop compounds showed a synergistic anti-inflammatory effect in combination with the curdlan-based biomaterial (Cur). This phenomenon is very interesting from a scientific and medical point of view and could be related to two scenarios. Firstly, the properties of the curdlan-based biomaterial should be considered. It was produced using ion-exchanging dialysis against a calcium chloride solution. Our previous research proved that this biomaterial has calcium ions in its structure, which are released in contact with liquids [4]. In the current study, the SEM and EDS analyses (Figure 3) indicated that the curdlan-based biomaterials modified with bioactive compounds from hop also possessed calcium ions in their structures. A few scientific reports have indicated that calcium ions may have anti-inflammatory properties and that they may act synergistically with substances that have anti-inflammatory properties [70,71,72]. The second scenario may be associated with the anti-inflammatory properties of the compounds derived from hop [73,74,75,76]. Thus, it seems that the in vitro anti-inflammatory activity of the curdlan-based biomaterials enriched with both CE and XN may have resulted from beneficial interactions between the curdlan-based biomaterial and bioactive hop compounds.

### 2.8. Preliminary In Vivo Experiments in Zebrafish Larvae Model

The zebrafish larvae caudal fin regeneration model is an excellent research approach for the evaluation of tissue regeneration, primarily due to its rapid regeneration time, easy live tracking, and the lack of serious effects on the larvae after amputation [77,78,79,80]. An additional advantage is the relatively simple design of the larval fin-fold, which consists of two layers of skin [78,79,80,81]. The zebrafish’s skin is composed of a periderm at the superficial layer, an epidermis bilayer in the middle, and a basal layer attached to the basement membrane [79]. The natural characteristics of zebrafish make it an excellent model for studying the mechanism of cutaneous wound healing. Interestingly, due to the similarity between the skin structure of humans and zebrafish, it has been considered as model for wound healing [80]. On the other hand, an amputation of zebrafish larvae’s caudal fin is associated with oxidative stress, which results in the generation of increased amounts of ROS and an intensified inflammatory response from immune cells [77]. Accordingly, the biomaterial extracts that showed the best in vitro properties were assigned for preliminary in vivo studies on a *Danio rerio* larvae model. In the first step, the effect of the extracts on the behavior of the *Danio rerio* larvae (locomotor activity) was determined, then their effect on the rate of the caudal fin regeneration was evaluated.

#### 2.8.1. The Influence of Biomaterials on Larval Locomotor Activity

**Figure 11:**
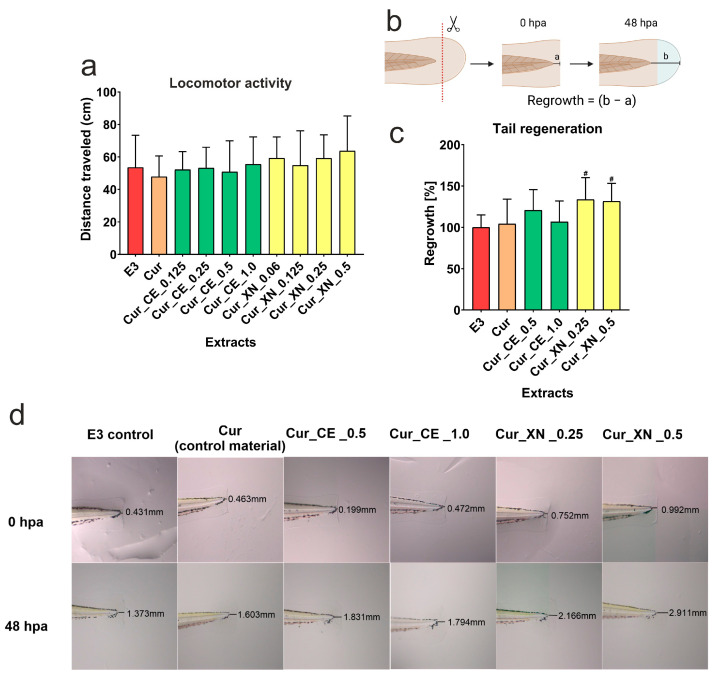
Total distance traveled in the darkness of <120 hpf zebrafish larvae after 48 h of incubation with selected extracts obtained from curdlan-based biomaterials enriched with CE or XN (**a**). No significance was found between tested groups, one-way ANOVA test followed by Tukey’s multiple comparisons, *p* > 0.05. Scheme of caudal fin amputation of 70 hpf larval zebrafish, and the post-amputation regrowth assessment after 48 h of incubation with selected extracts obtained from curdlan-based biomaterials enriched with CE or XN, a—length from the end point of the tail to the cut line, b—length from the end point of the tail to the end point of the the caudal fin regeneration after 48 h post amputation (**b**), created with BioRender.com. Caudal fin regrowth data are presented as normalized (%) to control E3 (**c**). ^#^ Significantly different results compared to results obtained for control E3; one-way ANOVA test followed by Tukey’s multiple comparisons, *p* < 0.05. Examples of stereoscopic microscope images showing regeneration of zebrafish larvae caudal fins after 48 h post-amputation (48 hpa) (**d**).

After 48 h of incubation, <120 dpf larval zebrafish did not exhibit any changes in their locomotor activity, expressed as the total distance traveled (Figure 11a). The results indicated that all the tested extracts obtained from the curdlan-based biomaterials enriched with CE or XN had no musculoskeletal- or neurological-dependent locomotion-related effects. Additionally, no morphological changes were observed in the macroscopic observations. Thus, based on the results, it was found that the tested extracts from the biomaterials were non-cytotoxic toward zebrafish larvae; therefore, they were subjected to further larval zebrafish testing. 

#### 2.8.2. The Influence of Biomaterials on Caudal Fin Regeneration

The results obtained in the wake of the assessment of the caudal fin regrowth indicated that both curdlan-based biomaterials enriched with XN (Cur_XN_0.25 and Cur_XN_0.5) significantly increased the regeneration of the amputated fins of the larval zebrafish (Figure 11b–d). The regrowth of the caudal fin was greater by averages of 33.6% and 31.5%, respectively, when compared to the E3 control. Neither Cur_CE_0.5 (an increase of 20.8%) nor Cur_CE_1.0 (6.8%) stimulated caudal fin regrowth in a statistically significant manner. These data indicated that the curdlan-based biomaterials enriched with xanthohumol showed regenerative properties in vivo.

Numerous studies have shown that zebrafish provide an in vivo research model for determining the usefulness of drugs and natural compounds related to wound healing [82]. Xie et al. demonstrated that a ginseng extract containing bioactive compounds (including Rg1, which exhibited anti-inflammatory and antioxidant properties when in contact with the wound) can accelerate amputated tail regeneration by increasing the rate of collagen synthesis at the wound site [83,84]. Similar conclusions were made by Raghupathy et al. [85], who evaluated the local application of a Curcuma longa extract (CLE) as a compound to promote the wound healing process. They demonstrated that CLE accelerated the regeneration process of the amputated fin in adult zebrafish. Moreover, Kim et al. showed that CLE possessed the ability to reduce oxidative stress induced by hydrogen peroxide (H_2_O_2_) in zebrafish embryos [86]. 

In this study, we proved that bioactive hop compounds (especially XN) are promising agents for the modification of wound dressings, as they support the wound healing process. Most probably, this phenomenon is associated with the antioxidant and anti-inflammatory activities of the investigated hop compounds—CE and XN (for more detail see Section 2.1). Moreover, we confirmed the observations provided by other authors, indicating that zebrafish is a beneficial model for the evaluation of wound healing and regenerative processes.

## 3. Materials and Methods

### 3.1. Materials

The curdlan powder from *Alcaligenes faecalis* var. *myxogenes* (cat. number 281-80531) was supplied by FUJIFILM Wako Pure Chemical Corporation (Hong Kong, Japan). The crude extract (CE) from the hop (α-acids: 42.48% *m/m* and β-acids: 19.07% *m/m* according to GC-MS analysis; terpenes and volatile compounds, mainly myrcene: 20.51%, humulene: 18.30%, β-caryophyllene: 4.88%, and β-farnesene: 4.69% according to U-HPLC analysis) was obtained using a dynamic supercritical fluid extraction process, according to the procedure described in detail previously [21] and provided by Lukasiewicz Research Network-New Chemical Synthesis Institute (Pulawy, Poland). Hop-RXn^TM^ powder (Xanthohumol—XN content over 99.0%) was purchased from BioActive-Tech Ltd. (Lublin, Poland). *Staphylococcus aureus* (ATCC 25923), *Staphylococcus epidermidis* (ATCC 12228), normal human skin fibroblasts (BJ cell line, ATCC^®^ CRL-2522^TM^), human acute monocytic leukemia cells (THP-1 cell line, ATCC^®^ TIB-202^TM^), and culture media were received from American Type Culture Collection (ATCC, Manassas, VA, USA), while Mueller Hinton broth (MHB) and Mueller Hinton agar (MHA) were from Oxoid Hampshire (Hampshire, UK). A Live/dead BacLight Bacterial Viability Kit and AlexaFluor^TM^ 635 Phalloidin dye were provided by ThermoFisher Scientific (Waltham, MA, USA). A COX (ovine/human) Inhibitor Screening Assay Kit and Lipoxygenase Inhibitor Screening Assay Kit were delivered by Cayman Chemical Company, Ann Arbor, MI, USA. Ascorbic acid, 2,2-diphenyl-1-picrylhydrazyl radical (DPPH^•^), 2,2′-azino-bis-(3-ethyl-benzothiazole-6-sulfonic acid) (ABTS^●+^), nordihydroguaiaretic acid (NDGA), indomethacin, ethylene-diaminetetraacetic acid, and the other reagents were purchased from Merck (Burlington, MA, USA). For all the analyses, Milli-Q water was used (Merck Millipore, Burlington, MA, USA).

### 3.2. Characterization of CE and XN

Firstly, two-fold serial dilutions of the CE and XN were prepared in a 0.3 M sodium hydroxide (NaOH) solution. The antioxidant properties of the CE and XN were determined using the 2,2-diphenyl-1-picryl-hydrazyl (DPPH^•^) free radical method and a 2,2′-azino-bis-(3-ethyl-benzothiazole-6-sulfonic acid) (ABTS^●+^) decolorization assay. These analyses were performed according to the protocols described by Szewczyk et al. [78]. In turn, the anti-inflammatory features (inhibitory activities of COX-1, COX-2, and lipoxygenase) of the CE and XN were assessed using commercially available ELISA tests, in accordance with the manufacturer’s guidelines.

### 3.3. Preparation of Curdlan-Based Biomaterials Improved with CE or XN

The curdlan-based biomaterials were produced according to the procedure described by us earlier [4], but with a modification involving the addition of bioactive hop compounds (CE or XN). Thus, the hop compounds were dissolved in a 0.3 M NaOH solution at concentrations of 0.125–16 μg/mL (CE) and 0.03125–4 μg/mL (XN)—these concentrations were selected based on preliminary antibacterial results (data not shown). Then, 1 mL of each solution was added to the curdlan powder (0.11 g). After the complete dissolution of the polymer, the resultant homogenous mixtures were placed in round-shaped molds and maintained at 4 °C overnight to remove air bubbles. The gelation process was carried out at room temperature by adding a 2% solution of calcium chloride. After 24 h, the hydrogels were removed from the molds, rinsed with water, frozen at −20 °C, and freeze-dried. The dry biomaterials were sterilized using ethylene oxide (55 °C, 3 h). For better clarity, the preparation process of the biomaterials is presented in Figure S1, while their names and compositions are presented in Table 1.

### 3.4. ATR-FTIR Analysis

The Fourier transform infrared spectroscopy (FTIR) absorbance spectra were acquired with the use of an IRSpirit (Shimadzu, Kyoto, Japan) equipped with a DLATGS detector. The measurements were performed in attenuated total reflectance (ATR) mode using the QATR™-S Single-Reflection Accessory with a diamond crystal (Shimadzu, Kyoto, Japan). Thin films of the curdlan and curdlan-based samples with hop extract (CE) and xanthohumol (XN) were prepared by compressing the obtained hydrogels using a 125 MPa compression pressure for 30 s and a manual hydraulic press (Specac, Orpington, UK). Then, the thin film samples were placed directly onto the crystal with a contact area diameter of 1.8 mm and pressed against its surface with a clamp mechanism. The spectra were recorded in the range of 4000–500 cm^−1^ with 36 scans at a resolution of 4 cm^−1^ from four locations for each film (one in the center of the film and three around the perimeter), and the resultant curves were averaged. In order to analyze the quantitative changes, the second derivatives in the specific regions were determined. The raw spectra were pre-processed using a rubberband baseline correction, a 15-point Savitzky-Golay (SG) smoothing, and vector normalization. Grams/AI 8.0 software (Thermo Electron Corporation; Waltham, MA, USA) was applied for the analysis of the recorded data. The Origin Pro 2021b (OriginLab Co., Northampton, MA, USA) was used for the graphical processing of the FTIR spectra and their second derivatives.

### 3.5. Microstructure Analysis

This analysis was performed using dry biomaterials, namely after the fabrication process. Before the analysis, the biomaterials were coated with an amorphous carbon layer to provide conductive surfaces (Sputter coater SCD 005/CEA 035, BAL-TEC, Balzers, Lichtenstein, Germany). The morphology of the biomaterials was tested using a scanning electron microscope (SEM) Gemini FESEM ULTRA PLUS (Carl ZEISS, Oberkochen, Germany) with an acceleration voltage of 3 kV. The quantitative X-ray microanalysis of the chemical elements content and distribution of the materials was performed using the energy-dispersive spectrometry (EDS) method with an X-ray spectrometer (Quantax 400, Bruker, Carteret, NJ, USA).

### 3.6. Contact Angle Measurements

The measurements of the contact angles were performed by placing a drop of deionized water with a conductivity of 0.2 µS/cm (HLP 20UV, Hydrolab, Straszyn, Poland) on the biomaterials’ surfaces. The drop shape was recorded directly using a digital camera and processed with the Krüss ADVANCE computer program (Krüss, Hamburg, Germany). The measurements were repeated three times for each sample. The average values and standard deviations (SDs) were calculated.

### 3.7. Evaluation of Wound Exudate Absorption Capacity

The ability of the biomaterials to absorb simulated wound fluid (SWF) was assessed based on the procedure described by us earlier [4]. This was established as growth in biomaterial weight (Wg) over time. 

### 3.8. Evaluation of Cell-Biomaterial Interaction In Vitro

All the experiments were performed using liquid extracts obtained from the biomaterials. The extracts were prepared in culture media (MHB for bacteria, EMEM for skin fibroblasts, and RPMI 1640 for THP-1-derived macrophages), according to the ISO 10993-5: 2009 standard [87] and ISO 10993-12:2012 standard [88]. The control extracts were culture media incubated without biomaterials. This precise procedure was described in detail earlier [3].

#### 3.8.1. Evaluation of Antibacterial Properties

The antibacterial properties of the biomaterials were assessed against *S. aureus* and *S. epidermidis*. The bacterial inoculums were prepared as described previously [89]. After 24 h of incubation with the liquid extracts, the optical densities (OD) of the bacterial suspensions were measured at 600 nm.

#### 3.8.2. Cytocompatibility towards Human Skin Fibroblasts

The influence of the biomaterial extracts on the viability of BJ fibroblasts was evaluated after 24 h of incubation using an MTT assay. In turn, the proliferation of the BJ cells was assessed after 2 and 4 days of incubation with the investigated extracts using an MTT assay. Additionally, after 4 days of incubation, nuclei/cytoskeleton staining was performed in order to visualize the cell morphology. The cells were observed using a confocal laser scanning microscope (CLSM, Olympus Fluoview equipped with FV1000, Shinjuku, Japan). The detailed procedure of this was described earlier [4].

#### 3.8.3. Inflammatory Activity of Human Macrophages

At the beginning of the experiment, THP-1 monocytes were differentiated into THP-1-derived macrophages via incubation with 200 nM phorbol 12-myristate 13-acetate (PMA). After 48 h of incubation, the stimulating medium was replaced with the liquid extracts obtained from the biomaterials and inflammation was induced by the addition of 100 ng/mL of lipopolysaccharide (LPS). Moreover, cells incubated with a culture medium without LPS served as a negative control (Control), while cells incubated with a culture medium with the presence of LPS were treated as a positive control (Control + LPS). After 24 h of incubation, the concentration of synthesized interleukin 6 (IL-6) was assessed via an ELISA test, according to the supplier’s protocol. 

### 3.9. Evaluation of Response of Zebrafish (Danio rerio) Larvae to Biomaterials

Preliminary in vivo studies were performed on zebrafish larvae, as they have been successfully used to evaluate host–biomaterial interactions [6,90,91,92]. In addition, the conduction of in vivo research using zebrafish is in good accordance with the 3Rs principle (replacement, reduction, and refinement), especially in the context of substituting mammals with the use of organisms of a lower taxonomic level [93]. Zebrafish embryos (*Danio rerio*, AB strain) were cultured under standard conditions (28.5 °C ± 0.5 °C, 14/10 h light/dark cycle, E3 medium) in the Experimental Medicine Centre, Medical University of Lublin (Poland) [94]. The fish were maintained in pursuance of the National Institute of Health Guidelines for the Care and use of Laboratory Animals and the European Community Council Directive for Care and Use of Laboratory Animals of 22 September 2010 (2010/63/EU). Females and males in a ratio of 1:2 were transferred to spawning tanks overnight. The next day/night cycle, at 10 a.m., the tank separators were removed and the fish started spawning. After this spawning, the eggs were transferred to Petri dishes (100 eggs/dish). For the experiments, 16 zebrafish larvae 70 h post-fertilization (hpf) were used (*n* = 16 per investigated group). Biomaterial extracts were obtained in the E3 medium according to the same procedure as that in the in vitro studies (for details, see Section 3.8). The E3 medium incubated without biomaterials served as a control extract (marked as E3 control). Upon the completion of the experiments, the larvae were humanely euthanized via a tricaine overdose (300–500 g/L). All the experiments were finalized in <120 hpf zebrafish larvae and were therefore exempt from ethical approval [95].

#### 3.9.1. Locomotor Behavior

Before the main experiments, the influence of the obtained extracts on the larval locomotor activity was tested in DanioVision™ by Noldus (Leesburg, VA, USA). In total, 70 hpf zebrafish larvae were transferred to 96-well plates (1 larva/well/200 μL extract or E3 control extract) and, after 48 h of incubation, they were evaluated using the EthoVision XT 17 video tracking software by Noldus. The plates were put into an observation chamber and, after 30 min of habituation in standard light conditions, the larvae were exposed to a 5 min period of extensive light (44.00 lux) followed by a 5 min period of complete darkness. The larval locomotor activity was expressed as the total distance travelled (cm) during the dark period. 

#### 3.9.2. Regeneration Process

The healing process of the zebrafish larvae (measured as the ability to regenerate the caudal fin after amputation) was carried out according to the general procedure described by Gu et al. [77], but with slight modifications. Briefly, the zebrafish larvae (70 hpf) were anesthetized with a 168 g/L tricaine solution and an amputation of their caudal fins posterior to the notochord was performed with a surgical scalpel under a Zeiss Stemi 508 stereomicroscope (Jena, Germany). The larvae were incubated in an E3 control extract or the biomaterial extracts. Moreover, the larvae that didn’t undergo an amputation procedure were incubated in the E3 medium and considered as an additional control of the experiment. Immediately after the amputation and after 48 h post-amputation procedure (48 hpa), the fins were imaged under a Zeiss Axio Vert stereomicroscope. The caudal fin growth (i.e., percentage of regenerated fin area) was established using ImageJ 1.52v software (Maryland, MD, USA). The results were normalized to the control group.

### 3.10. Statistical Analysis

At least three separate replicates were performed during the experiments. The results were provided as mean values ± standard deviation (SD). Before the statistical comparison, a D’Agostino and Pearson omnibus normality test was performed in order to determine the normal distribution of the data. Then, a one-way ANOVA test, followed by Tukey’s multiple comparison tests or a two-way ANOVA test, followed by a Bonferroni comparison test, were applied to set up the statistical differences between the tested groups (*p* < 0.05), (GraphPad Prism 5, Version 5.04 Software, Boston, MA, USA).

## 4. Conclusions

In this work, the structural, physicochemical, and biological properties of biomaterials enriched with bioactive compounds extracted from hops were evaluated. The results showed that curdlan-based hydrogels modified with bioactive compounds derived from the hop, namely crude extract (CE) rich in α-acids, β-acids, and essential oils or xanthohumol (XN), demonstrated a wide potential for practical biomedical application. The produced biomaterials were characterized by a stable structure, regardless of the concentration of the incorporated bioactive compound, and their elemental composition did not change. The efficient incorporation of these bioactive hop compounds into the curdlan-based hydrogel matrix was demonstrated. The ATR-FTIR analysis revealed the presence of hydrogen bonds between the curdlan matrix and CE or XN. As the concentration of the incorporated bioactive compound increased, there was a reduction in the free hydrophilic sites in the biomaterials (resulting from the presence of hydrogen bonds), which, in turn, reduced their porosity, wettability, and absorbent capacity compared to the control material. Nevertheless, all these parameters were still satisfactory. Among the tested curdlan-based biomaterials, Cur_XN_0.5 seems the most promising for potential applications in the treatment of chronic wounds. The performed in vitro tests indicated that the extract obtained from the fabricated biomaterial did not possess significant antibacterial properties, while it did not exhibit cytotoxicity towards human skin fibroblasts, as well as not inhibiting their proliferation. Moreover, this extract had the ability to reduce the IL-6 production by human macrophages after LPS stimulation compared to the control, indicating its anti-inflammatory activity. Most important, the in vivo studies conducted using a model organism of *Danio rerio* larvae showed that the tested extract not only possessed no negative effects on the musculoskeletal and nervous systems, but also significantly increased the regeneration of the amputated caudal fin. These phenomena are most likely associated with the high antioxidant and anti-inflammatory properties of xanthohumol. This manuscript provided new data on the biomedical application of hop compounds, as this is the first time that the incorporation of bioactive hop compounds into a biopolymer matrix for regenerative medicine applications has been described. The obtained results appear to be a promising step forward in the development of new dressings of 100% natural origin for the treatment of chronic wounds, such as diabetic foot ulcers, venous leg ulcers, or pressure ulcers. Nevertheless, this paper presents preliminary results and addition in vivo experiments are needed in order to precisely determine the beneficial effect of the Cur_XN_0.5 biomaterial on the wound healing process. In future, we plan to expand our research involving the evaluation of the wound healing, anti-inflammatory, and pro-angiogenic properties of such biomaterials in animal models with chronic wounds. The scientific challenge for the future is also to undertake work to increase the antibacterial properties of such biomaterials, while maintaining their antioxidant, anti-inflammatory, and cytocompatible properties.

## 5. Patents

The general fabrication procedure of the curdlan dressings was described in Polish patent no. 238256 (“The fabrication method of absorbent biomaterial based on curdlan for biomedical applications”).

## Figures and Tables

**Table 1:** The composition of produced curdlan-based biomaterials improved with hop components.

Biomaterial Code	Concentration of Curdlan (Cur) [wt.%]	Concentration of Crude Extract (CE) [μg/mL]	Concentration of Xanthohumol (XN) [μg/mL]
Cur	11	-	-
Cur_CE_0.125	11	0.125	-
Cur_CE_0.25	11	0.25	-
Cur_CE_0.5	11	0.5	-
Cur_CE_1	11	1	-
Cur_CE_2	11	2	-
Cur_CE_4	11	4	-
Cur_CE_8	11	8	-
Cur_CE_16	11	16	-
Cur_XN_0.0156	11	-	0.0156
Cur_XN_0.0313	11	-	0.03123
Cur_XN_0.0625	11	-	0.0625
Cur_XN_0.125	11	-	0.125
Cur_XN_0.25	11	-	0.25
Cur_XN_0.5	11	-	0.5
Cur_XN_1	11	-	1
Cur_XN_2	11	-	2

## Data Availability

Data are available on reasonable request.

## Supplementary Materials

The following supporting information can be downloaded at: https://www.mdpi.com/article/10.3390/ijms241210295/s1.
