# Supplementary material for: Do Curdlan Hydrogels Improved with Bioactive Compounds from Hop Exhibit Beneficial Properties for Skin Wound Healing?
Source: Int J Mol Sci. 2023 Jun 18;24(12):10295. doi: 10.3390/ijms241210295 (PMC10299105; doi:10.3390/ijms241210295)
Supplement: Supplementary file 1 [file ijms-24-10295-s001.zip › ijms-2438669-supplementary.pdf]

Supplementary Data

Do Curdlan Hydrogels Improved With Bioactive Compounds From Hop Exhibit Beneficial Properties In The Context Of Skin Wound Healing?

Aleksandra Nurzynska [1,*], Katarzyna Klimek [1], Agnieszka Michalak [2], Katarzyna Dos Santos Szewczyk [3], Marta Arczewska [4,5], Urszula Szalaj [6,7], Mariusz Gagos [8,9] and Grazyna Ginalska [1]

1 Medical University of Lublin, Chair and Department of Biochemistry and Biotechnology, Chodzki 1 Street, 20-093 Lublin, Poland
2 Medical University of Lublin, Independent Laboratory of Behavioral Studies, Chodzki 4 a Street, 20-093 Lublin, Poland
3 Medical University of Lublin, Department of Pharmaceutical Botany, Chodzki 1 Street, 20-093 Lublin, Poland
4 University of Life Sciences, Department of Biophysics, Akademicka 13 Street, 20-033 Lublin, Poland;
5 Medical University of Lublin, Department of Biophysics, Jaczewskiego 4 Street, 20-090 Lublin, Poland
6 Polish Academy of Science, Laboratory of Nanostructures, Sokolowska 29/37 Street, 01-142 Warsaw, Poland
7 Faculty of Materials Engineering, Warsaw University of Technology, Warsaw, Poland
8 Maria Curie-Sklodowska University, Department of Cell Biology, Akademicka 19, 20-033 Lublin, Poland
9 Medical University of Lublin, Department of Biochemistry and Molecular Biology, Chodzki Street 1, 20-093 Lublin;
* Correspondence: aleksandra.nurzynska@umlub.pl

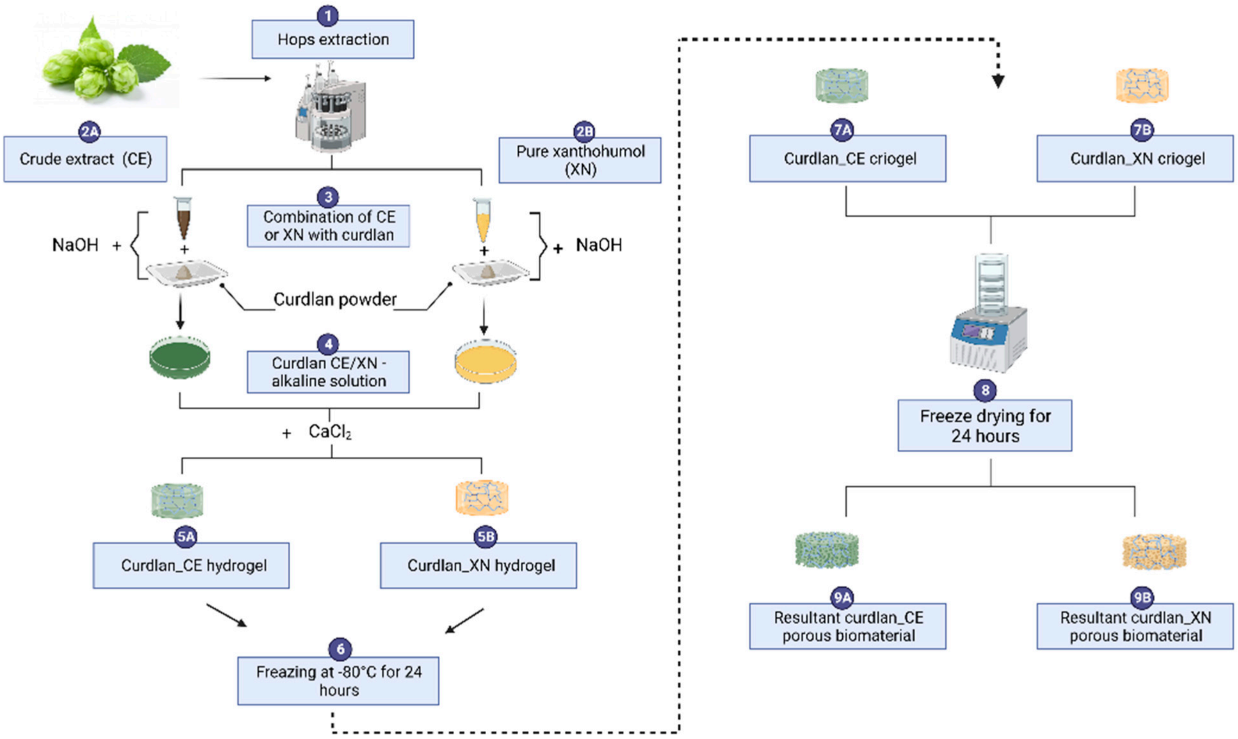

Figure S1. Schematic illustration showing the fabrication method of curdlan-based biomaterials modified with bioactive hop compounds.

Table S1. *In vitro* antioxidant properties of the crude extract (CE) and xanthohumol (XN) evaluated using DPPH and $ABTS^{\bullet +}$ assays. Ascorbic acid (AA) was used as positive control (standard).

IC_{50} ± SD [mg/mL]		
**Sample**	**DPPH assay**	**$ABTS^{\bullet +}$ assay**
CE	0.606 ± 0.011	0.486 ± 0.001
XN	5.467 ± 0.065	0.638 ± 0.023
AA	0.478 ± 0.02	1.988 ± 0.092

Table S2. *In vitro* anti-inflammatory properties of the crude extract (CE) and xanthohumol (XN) evaluated using cyclooxygenase-1 (COX-1), cyclooxygenase-2 (COX-2), and lipoxygenase (LOX) inhibitory assays. Indomethacin (IND) and nordihydroguaiaretic acid (NDGA) were used as positive controls (standards).

IC_{50} ± SD [μg/mL]			
**Sample**	**COX-1 Inhibition**	**COX-2 Inhibition**	**LOX Inhibition**
CE	67.89 ± 0.78	56.92 ± 0.43	36.80 ± 0.54
XN	20.54 ± 1.18	17.15 ± 0.28	11.13 ± 0.18
IND	4.09 ± 0.04	3.57 ± 0.03	-
NDGA	-	-	5.47 ± 0.11
